# Erythropoietin Action in Stress Response, Tissue Maintenance and Metabolism

**DOI:** 10.3390/ijms150610296

**Published:** 2014-06-10

**Authors:** Yuanyuan Zhang, Li Wang, Soumyadeep Dey, Mawadda Alnaeeli, Sukanya Suresh, Heather Rogers, Ruifeng Teng, Constance Tom Noguchi

**Affiliations:** 1 Molecular Medicine Branch, National Institute of Diabetes and Digestive and Kidney Diseases, National Institutes of Health, Bethesda, MD 20892, USA; E-Mails: yuanyuan.zhang2@nih.gov (Y.Z.); shoumo.dey@nih.gov (S.D.); sukanya.suresh@nih.gov (S.S.); heatherro@niddk.nih.gov (H.R.); 2 Faculty of Health Sciences, University of Macau, Macau SAR, China; E-Mail: liwang@umac.mo; 3 Department of Biological Sciences, Ohio University, Zanesville, OH 43701, USA; E-Mail: al-naeel@ohio.edu; 4 Diabetes Institute, Ohio University, Athens, OH 45701, USA; 5 Mouse Metabolism Core Laboratory, National Institute of Diabetes and Digestive and Kidney Diseases, National Institutes of Health, Bethesda, MD 20892, USA; E-Mail: tengr@niddk.nih.gov

**Keywords:** erythropoietin, erythropoietin receptor, signal transduction, stress response, wound healing, endothelial, brain, cardiovascular, inflammation, metabolism, obesity

## Abstract

Erythropoietin (EPO) regulation of red blood cell production and its induction at reduced oxygen tension provides for the important erythropoietic response to ischemic stress. The cloning and production of recombinant human EPO has led to its clinical use in patients with anemia for two and half decades and has facilitated studies of EPO action. Reports of animal and cell models of ischemic stress *in vitro* and injury suggest potential EPO benefit beyond red blood cell production including vascular endothelial response to increase nitric oxide production, which facilitates oxygen delivery to brain, heart and other non-hematopoietic tissues. This review discusses these and other reports of EPO action beyond red blood cell production, including EPO response affecting metabolism and obesity in animal models. Observations of EPO activity in cell and animal model systems, including mice with tissue specific deletion of EPO receptor (EpoR), suggest the potential for EPO response in metabolism and disease.

## 1. Introduction

**Figure 1:**
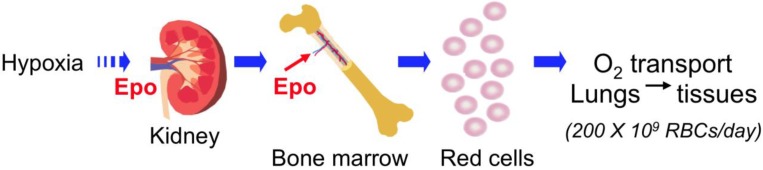
Erythropoietin (EPO) is required for red blood cell production. EPO is produced in the kidney in a hypoxia dependent manner and is secreted into the circulation. EPO is the primary regulator for production of red blood cells that transport oxygen in the circulation from the lungs to the tissues. In the bone marrow, EPO stimulates erythroid progenitor cell survival, proliferation, and differentiation to produce 200 billion new red blood cells daily to compensate for the red blood cell lifespan of 120 days.

The cytokine erythropoietin (EPO) is required for production of red blood cells. In the human body, EPO stimulates the daily production of about 200 billion new red blood cells to compensate for the limited red blood cell lifespan of 110–120 days (Figure 1). EPO is produced primarily in the kidney and is secreted into the blood circulatory system and targets erythroid progenitor cells in the bone marrow to stimulate red blood cell production (erythropoiesis). The protein content of red blood cells is 95% hemoglobin, which binds oxygen cooperatively for transport from the lungs for delivery to the tissues. Hence, the primary function of EPO is to regulate oxygen delivery via the production of red blood cells and is facilitated by the hypoxia induction of *EPO* gene transcription resulting in sensitivity of EPO production to the local oxygen environment [1]. EPO acts by binding to its specific receptor on the surface of erythroid progenitor cells to stimulate cell survival, proliferation and differentiation. Knockout of EPO (*EPO^−^*^/*−*^) or EPO receptor (*EpoR^−^*^/*−*^) in mice results in embryonic death due to severe anemia [2,3]. This review will discuss reports of EPO activity in non-erythroid systems on tissue maintenance and stress response in animal models of ischemic and traumatic injury and metabolism. The non-erythroid activity and cytoprotective potential of EPO have not yet been confirmed in placebo controlled clinical trials, which is reviewed elsewhere [4,5].

## 2. Erythropoietin (EPO) Regulation

EPO, a member of the hematopoietic cytokine superfamily with structural similarity to growth hormone, is encoded as a 193 amino acid polypeptide containing a 27 amino acid canonical leader sequence and a carboxyl terminal arginine that is removed by posttranslational cleavage [6]. The resultant 165 amino acid glycoprotein consists of four α-helices and two disulfide bonds with three asparagine *N*-glycosylation sites and one serine *O*-glycosylation site linking up to 40% or more carbohydrate to extend its *in vivo* half-life [7,8,9]. Reduced oxygen tension induces EPO concentration in the local microenvironment or, when produced in the kidney and secreted, in the circulating blood [1].

### 2.1. EPO Production in Fetal Liver and Adult Kidney

During development, EPO is mainly produced in the fetal liver by hepatocytes [10,11]. As the site of erythropoiesis changes from the fetal liver during development to the bone marrow at birth, EPO production switches from the fetal liver to the kidney. EPO expression in the kidney was observed by 17 weeks in human gestation [10]. The cells producing EPO in the kidney are the peritubular interstitial fibroblasts [12,13,14]. Reduced oxygen availability significantly induces *EPO* gene expression in fetal liver, adult liver and kidney [12,15,16]. The hypoxic induction of EPO production of up to 150-fold or more in the kidney results from an increase in the number of EPO-producing cells rather than from an increase in EPO production per cell [12,17].

### 2.2. Regulation of EPO Gene Expression by Hypoxia Inducible Factor (HIF)

EPO production is regulated primarily by gene transcription at the cellular level by multiple transcription factors, such as hypoxia induced factor (HIF), prolyl hydroxylase domain-containing protein (PHD), and GATA binding protein (GATA) [1]. The *EPO* gene contains a 3' enhancer identified as the hypoxia-responsive element (HRE) in human hepatoma cells [18,19]. HIF contains a constitutive β-unit (aryl hydrocarbon receptor nuclear translocator (ARNT)) in the nucleus and an α-subunit in the cytoplasm [1]. The α-subunit of HIF contains two highly conserved LXXLAP sequences, which can be recognized by an oxygen- and iron-dependent prolyl hydroxylase, leading to the conversion of proline into hydroxyproline. This conversion is necessary and sufficient for the binding of von Hippel-Lindau (vHL) protein, docking of ubiquitin E3 ligase and degradation of the α-subunit. Oxygen is required for the prolyl hydroxylase catalysis of the HIF α-subunit and its enzymatic rate is reduced at low oxygen, providing for oxygen regulation of HIFα stabilization, transport to the nucleus, dimerization with ARNT and activation of gene expression such as hypoxia responsive vascular endothelial growth factor (*VEGF*), glucose transporter 1 (*GLUT1*), and *EPO* [1]. HIF-1α preferentially activates VEGF and GLUT1, while HIF-2α plays a critical role in the regulation of EPO production [20,21]. The hydroxylation of HIFα is carried out by three prolyl-4-hydroxylase domain proteins (PHD1, PHD2 and PHD3), among which PHD2 plays the predominant role at most sites. Human genetic mutations showed that disruption of PHD2 or vHL proteins has been identified as causes for abnormally high levels of EPO production that results in polycythemia [22,23].

### 2.3. EPO Regulation by Other Transcription Factors

The GATA transcription factors that contribute importantly to erythropoiesis, such as GATA1 and GATA2, also contribute to *EPO* gene regulation [12]. GATAs belong to the zinc finger protein family. GATA1, GATA2 and GATA3 are primarily involved in hematopoiesis although their expression is also seen in some non-hematopoietic tissues such as kidney, urinary tract and nervous system, while GATA4, GATA5 and GATA6 are found in the heart, gut and extraembryonic endoderm. GATA1–3 have been reported to negatively regulate *EPO* gene expression through a promoter proximal GATA site [24,25], but GATA4 exhibits the most prominent *EPO* promoter binding activity *in vitro* and *in vivo*, which activates *EPO* gene expression in fetal liver and may participate in the switch of *EPO* expression from fetal liver to adult kidney [26]. EPO, in turn, can contribute to regulation of the *GATA* factors in erythroid, neural and skeletal myoblasts that also affects *EpoR* expression [27,28,29,30].

Other transcription factors, such as hepatic nuclear factor 4 (HNF4), retinoic X receptor-α (RXR-α), Wilms tumor suppressor (WT1), SMAD3 and Sp1, a member of the Sp/KLF family of transcription factors, all contribute to the regulation of *EPO* expression. HNF4 binds to the *EPO* gene 3' enhancer adjacent to the HIF-binding site [18,31]. HNF4 and HIF form a complex with transcription coactivator p300 (EP300) for the activation of stimulus-specific and tissue-specific gene transcription [32]. RXR-α competes with HNF4 for occupancy in the 3' *EPO* enhancer, contributing to *EPO* activation in the fetal liver during early definitive erythropoiesis; it is not necessary later in development as HNF4 activity in the fetal liver increases [33]. WT1 directly regulates *EPO* expression in hepatocytes and knockout of *Wt1* in mice decreases erythropoiesis in fetal liver [34,35]. SMAD3 binding to the 3' *EPO* enhancer, and Sp1 binding to the *EPO* promoter region also cooperate with HIF, HNF4 and p300 in *EPO* gene transcription and induction by hypoxia [36]. Together these factors act to stabilize the multifactorial complex interacting with the 3' enhancer and reinforce the promoter–enhancer contact.

## 3. EPO Receptor Expression in Erythroid Cells

Cloning of *EpoR*, first carried out by expression screening of a murine erythroleukemia (MEL) cell library in the monkey kidney fibroblast-like COS cells [37], facilitated quantification of *EpoR* gene expression. *EpoR* is expressed at the highest level on erythroid progenitor cells and provides for the critical regulation by EPO of red blood cell production. Expression of the mature erythroid form of *EpoR* beyond hematopoietic cells raises the possibility that EPO activity associated with survival, proliferation and differentiation of erythroid cells may not be restricted to erythropoiesis.

EPO, required for survival of erythroid progenitor cells, also stimulates their proliferation and differentiation, and acts through binding to its receptor on the surface of erythroid progenitor cells [2,3]. EPO signaling in hematopoietic cells relies not only on the EPO concentration but also on the level of EpoR cell-surface expression. The extent of EpoR expression largely determines the erythropoietic response. EpoR is expressed in a cell-restricted fashion and expression depends on the stage of differentiation. EpoR expresses at a relatively low level on early erythroid progenitor cells or burst forming unit-erythroid (BFU-E) stage [38]. EpoR is then increased during erythroid differentiation by more than 10-fold on erythroid progenitor cells by the colony forming unit-erythroid (CFU-E) stage, which requires EPO signaling for protection against apoptosis for survival. During late erythropoiesis, EpoR expression is down-regulated and EPO/EpoR signaling is no longer required for erythroid cell survival [38]. Interestingly, EpoR expression is up regulated by EPO stimulation of erythroid cells via EPO induction of erythroid transcription factors [28]. *EpoR* gene transcription is regulated by erythroid specific transcription factors including GATA1 and T-cell acute lymphocytic leukemia 1 (TAL1) [39,40,41]. These erythroid transcription factors including erythroid Kruppel-like factor (EKLF or KLF1) are induced by EPO stimulation, which provides a mechanism for EPO regulating the expression of its own receptor.

**Figure 2:**
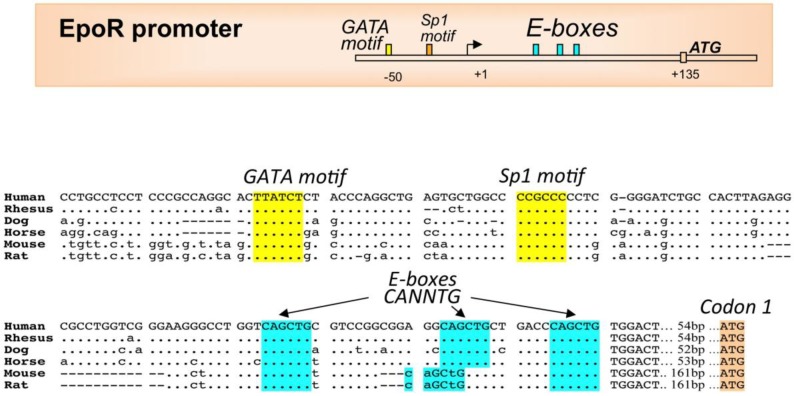
*EpoR* gene proximal promoter region with conserved regulatory protein binding sites. **Top panel**, schematic of the human EpoR proximal promoter region extending to the translation start site (ATG, +135) showing conserved GATA binding motif (AGATAA), a Sp1 binding motif (CCGCCC) upstream of the transcription start site and 3-E-boxes (CAGCTG) downstream in the 5' untranslated transcribed region. The E-boxes are consensus-binding sites for bHLH transcription factors. **Middle and bottom panels**, homologous mammalian sequences showing alignment of conserved transcription factor binding motifs.

Sequence conservation in the *EpoR* gene proximal promoter includes the GATA1 and Sp1 binding motifs that are required for high level *EpoR* gene activation and extends 3' beyond the transcription start site to include a 37-bp region with 3 E-box motifs located downstream from the GATA1 binding site by 60 and 75 bps, respectively, in the mouse and human genomes (Figure 2). *EpoR* gene expression in erythroid cells is regulated not only by GATA transcription factor (mainly GATA1) but also by Sp1 [41]. TAL1 binds to the E-box motifs and recruits GATA1 to the GATA site via forming complex with LIM domain only 2 (LMO2) and LIM domain-binding protein 1 (LDB1) [42]. *EpoR* transcription has been reported in non-erythroid cells such as neural cells, myoblasts but not mature muscle, and endothelial cells [29,43,44] with the GATA site and E-box motifs also contributing to active *EpoR* expression in these non-erythroid cell types [45,46].

## 4. EPO Signal Transduction

**Figure 3:**
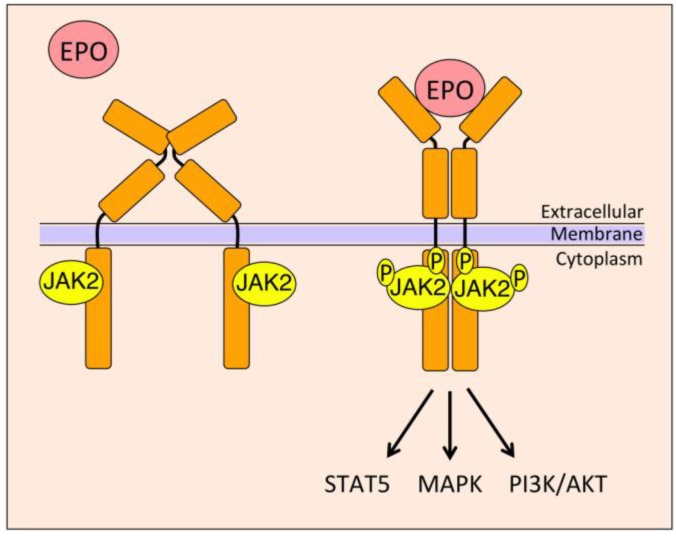
Model of erythroid EPO/EpoR signaling. The homodimeric EpoR molecule with Janus kinase 2 (JAK2) bound to its cytoplasmic domain is primed for EPO stimulation. EPO binding to the extracellular domain of EpoR results in conformational changes that bring JAK2 bound sites in close proximity to each other. This results in transphosphorylation of JAK2 and activation, which in turn increases other downstream signaling through signal transducer and activator of transcription 5 (STAT5), mitogen-activated protein kinase (MAPK) and phosphoinositide 3-kinase (PI3K)/AKT pathways.

EpoR is a type 1 cytokine receptor that consists of an extracellular domain with a conserved Trp-Ser-X-Trp-Ser (WSXWS) sequence above the transmembrane domain and does not contain an intrinsic tyrosine kinase domain for downstream signaling; instead it depends on Janus kinase 2 (JAK2), a non-receptor tyrosine kinase, which in its active form binds the Box1/Box2 region of EpoR [47]. EPO binding to the EpoR homodimer triggers a conformational change in the receptor cytoplasmic domain, bringing the JAK2 proteins in close proximity to each other and resulting in transphosphorylation and activation of JAK2 and EpoR [48] (Figure 3). EpoR activation initiates downstream cascades via different signaling pathways including signal transducer and activator of transcription (STAT), phosphoinositide 3-kinase (PI3K)/AKT, and mitogen-activated protein kinase (MAPK) via adapter proteins like Src homology containing protein (SHC) [49,50]. Activated EpoR provides phosphorylated tyrosine residues as docking sites for STATs and other Src homology 2 (SH2) domain proteins that lead to the activation of PI3K/AKT and MAPK signaling pathways. Recent evidence suggests an instructive role for EPO in determining hematopoietic progenitor cell fate. EPO suppresses non-erythroid lineage determination with no increase in early erythroid progenitor cells and, particularly at high dose, increases committed erythroid progenitors and decreases committed granulocyte/macrophage and megakaryocyte progenitors and suppresses lymphoid colony-forming cells, mediated through transcription reprogramming involving, in part, GATA-1, PI3K activation for erythroid gene induction and ERK activation for myeloid gene suppression [51].

### 4.1. Janus Kinase 2 (JAK2)/Signal Transducer and Activator of Transcription 5 (STAT5)

JAK2/STAT5 is the classical pathway activated in erythroid cells [52]. Activation of JAK2 induces the direct binding of STAT5 to EpoR cytoplasmic domain and subsequent phosphorylation by JAK2, which leads to STAT5 dimerization and translocation to the nucleus, where it binds DNA in a sequence-specific manner to drive gene transcription [53]. EPO-induced JAK2/STAT5 pathway up-regulates anti-apoptotic protein Bcl-x_L_. Unlike mice that lack either EPO or EpoR resulting in death *in utero*, double knockout of both STAT5A and STAT5B is not embryonic lethal and, rather, results in fetal anemia and defective response in adult mice to erythroid stress [54].

### 4.2. Mitogen-Activated Protein Kinase (MAPK)

The MAPK signaling pathway involves the tyrosine phosphorylation of SH2 inositol 5-phosphatase 1 (SHIP1), which binds to EpoR and recruits adapter proteins like SHC and growth factor receptor-bound protein 2 (GRB2) to the receptor [55]. This leads to downstream pathway activation of RAS/RAF, MEK and MAPK signaling. However, inhibition of RAF-1 activation did not alter EPO stimulated phosphorylation of MYC [56], suggesting other redundant pathways are also involved. In human erythroid colony forming cells, MAPK appears to be necessary for erythropoiesis [57], whereas in Friend murine erythroleukemia cells (MEL), MAPK inhibition induces erythroid differentiation [58]. Moreover, a mutant EpoR that lacks tyrosine phosphorylation sites in the carboxy-terminal region provided EPO-induced proliferation without activation of MAPK [59].

### 4.3. Phosphoinositide 3-Kinase (PI3K)

Activation of the PI3K pathway involves phosphorylation of phosphatidylinositol 4,5-bisphosphate (PI-(4,5)-P_2_), which then activates phosphoinositide-dependent kinase 1 (PDK1) leading to phosphorylation of AKT, a serine-threonine kinase. Activated AKT phosphorylates and inactivates BAD, forkhead transcription factor and caspase-9 to inhibit apoptosis [60,61]. Moreover, AKT phosphorylates and activates glycogen synthase kinase-3 (GSK-3), which regulates cyclin D1 and c-MYC [62]. EpoR activation also leads to its association with the SH2 domains of the PI3K regulatory subunit p85 [63,64]. p85 and SHP2 docks with phosphorylated Gab1 and Gab2 after EPO stimulation [65]. EPO stimulation has also been observed to induce phosphorylation of Vav protein leading to its association with EpoR and p85 [66]. PI3K activity is required for EPO induced initiation of c-MYC transcription whereas the MAPK pathway is required for its elongation [56]. PI3K signaling is also involved in the EPO-dependent activation of ribosomal protein S6 kinase p70S6k [67].

### 4.4. Signal Transducer and Activator of Transcription 3 (STAT3)

In erythroid cells, while EPO stimulates primarily JAK2/STAT5 activation, STAT3 can also be phosphorylated, albeit to a lesser extent than STAT5, and may act to provide limited erythropoietic activity in mice with loss of STAT5 [54,68]. In brain, the protective effects of EPO are mediated via STAT3 activation, which promotes increased Bcl-2 and Bcl-x_L_ in animal models of experimental intracerebral hemorrhage, and traumatic brain injury, and facilitates neuritogenesis [69,70,71]. EPO treatment also stimulates STAT3 activation in macrophages and reduces macrophage infiltration in white adipose tissue in a mouse model of diet induced obesity [72].

## 5. EPO Action in Non-Hematopoietic Tissue

Availability of recombinant EPO following the cloning of the gene provided an important reagent for evaluation of EPO activity in non-hematopoietic tissue including endothelial and neural cells. Reports of EPO binding, EPO response, *EpoR* mRNA and/or EpoR protein expression beyond erythropoiesis suggested the possibility of EPO action in non-hematopoietic cells or tissues in model systems. These observations are not without controversy [73,74] and concerns were raised about the functional significance of *EpoR* transcripts detected at low level in tissues reported to exhibit EPO response and specificity of commercial EpoR antibodies [74].

### 5.1. Endothelial Response

Endothelial cells from cesarean section-derived HUVECs were the first non-hematopoietic cell type reported to bind EPO, exhibit a proliferative response to EPO *in vitro*, and express the erythroid form of EpoR [43,75]. EPO was observed to protect cultures of rat cerebral microvascular endothelial cells from anoxia-induced injury by activating AKT1 and maintaining mitochondrial membrane potential and prevent apoptotic injury during oxidant stress through sirtuin 1 (SIRT1) pathways [76,77]. An important function of endothelial cells is to express endothelial nitric oxide (NO) synthase (eNOS/NOS3), which produces NO to regulate vascular tone and blood flow. The combination of reduced oxygen and EPO treatment in endothelial cells was observed to increase *EpoR* mRNA and protein expression, modulate eNOS expression and stimulate NO production [78,79]. In contrast, cell cultures at normal oxygen tension were reported to exhibit low *EpoR* mRNA expression, little protein in HUVEC in Western blotting using an N-terminus EpoR antibody, no EPO binding and no EPO stimulated AKT activation [74]. In animal models, mice expressing high level of transgenic EPO (*tg6*) with high hematocrit did not develop hypertension due to markedly increased levels of eNOS and NO in vascular tissue and in the circulation, and EPO treatment in rodents was also observed to stimulate NO production [80,81,82]. EPO is the premier erythroid cytokine that provides for up-regulation of mature red blood cell production in response to hypoxic or ischemic stress [1]. Increasing red cell number increases the oxygen carrying capacity of blood to enhance oxygen delivery to the tissues. However, EPO stimulation of erythroid progenitor cells in the bone marrow to increase survival, proliferation and differentiation results in a delay in the increase in red blood cell number following the onset of hypoxia. An increase in NO production would provide an acute response to increase oxygen delivery via regulation of vascular tone.

During development, *EpoR^−^*^/*−*^ mice exhibit severely affected vessel formation and decreased complexity of vessel networks [83]. However, mice with EpoR restricted to erythroid tissue do not show gross morphologic defects in the cardiovascular system, although they are more susceptible to pulmonary hypertension and pulmonary vascular remodeling [84,85]. In a model of hypoxia-induced pulmonary hypertension, these mice were reported to exhibit impaired endothelial cell mobilization and recruitment to pulmonary endothelium and lack hypoxia activation of eNOS in lungs observed in wild type mice; pulmonary hypertension was ameliorated by wild type bone marrow transplantation [85]. Beneficial effects mediated via EPO stimulation of endothelial cells including NO production have been suggested by EPO response observed in various animal injury or disease models in heart and brain [86,87,88]. In a rat model of chronic renal disease progression, EPO treatment reportedly activated AKT and eNOS, and reduced progressive vascular injury and organ failure [89]. In the mouse uterus, estrogen dependent EPO production was suggested to contribute to regulation of the cyclic changes in female reproductive organs by promoting growth of uterine tissues and angiogenesis in the endometrium [90]. However, it was also reported that estradiol treatment in female rats inhibits hypoxia induction of EPO, ovariectomized female rats increased hypoxia induction of EPO equal to males, and *in vitro* estrogen interfered with hypoxic induction of HIF and EPO in *in vitro* cultures [91].

### 5.2. EPO and Heart

During mouse development, *EpoR^−^*^/*−*^ mice show ventricular hyperplasia due to ineffective cell proliferation and expansion of myocardium [45,92]. Nevertheless, rescue of the *EpoR^−^*^/*−*^ phenotype using an erythroid specific *EpoR* transgene results in mice that survive to adulthood without gross morphological defects including the heart [84]. In contrast to increased susceptability to hypoxia induced pulmonary hypertension in these mice [85], they do not exhibit increased damage in ischemic-reperfusion injury in heart at 5 months of age [87], providing evidence that endogenous EPO is not cardioprotective.

In adult animal model studies, EPO treatment was observed to reduce cardiac ischemia/reperfusion injury, attributed in part to stimulation of NO production and as an acute response prior to increase in hematocrit [80,87], although EpoR was not detected in human heart and EPO signaling was not detected in rat neonatal cardiac myocytes [74]. EPO induced NO production by vascular endothelial cells is suggested to be mediated primarily by induction and activation of eNOS, particularly at reduced oxygen [78,93]. EPO stimulated eNOS activation was reported to be linked to PI3K signaling and the EPO associated reduction in cardiac injury was not observed in *eNOS^−^*^/*−*^ mice [87,94,95]. Furthermore, mice with ectopic transgenic EPO expression exhibited elevated eNOS activity and plasma NO level that prevented cardiovascular disease such as hypertension and thromboembolism and inhibition of NO synthase resulted in cardiovascular dysfunction and death [80]. Although it has been proposed that EPO reduction of cardiac ischemia/reperfusion injury is mediated by eNOS activity via NO secretion by cardiomyocytes [94], EPO treatment in mice with endogenous *EpoR* expression restricted largely to hematopoietic and endothelial cells exhibited stimulated eNOS activation in endothelial cells and reduced ischemic reperfusion injury similar to wild type control mice [87]. Hence, eNOS activation and endothelial response is suggested to be sufficient for the observed EPO activity in cardiac ischemia/reperfusion injury [87].

### 5.3. Brain and Neural Protection

During embryonic development, *EpoR* mRNA expression in mouse brain was observed to be high during mid-gestation coinciding with regions of neurogenesis in the developing embryo [45,96], and decreased with development, dropping by two-orders of magnitude from day E10 to birth [97]. Reports of EPO production in brain on the other side of the blood–brain barrier by astrocytes and neurons, found that EPO levels in the central nervous system did not follow circulatory levels and suggested that EPO is produced on both sides of the blood-brain barrier [98,99,100]. As in the kidney, EPO production in the brain was observed to be hypoxia-inducible in rodent and primate brains [101] and to increase more than 100-fold in astrocytes incubated under hypoxic conditions [98,99,102]. The reported changing pattern of EpoR and EPO expression in the developing mouse brain including the neural tube, optic placode and forebrain suggested a delay of 0.5–1 days, with tissue remodeling and associated apoptosis in the subset of neurons that lack EPO [103,104,105]. Furthermore, it was observed that reduced oxygen increased *EpoR* mRNA expression in neural cells, and EPO binding in rodent and primate brains [45,98,101,106] that persist for 24 h or more [101,107,108]. Hypoxia induced EPO production and EPO treatment were suggested to be associated with neural progenitor cell proliferation and differentiation [109,110]. In the absence of neurotrophic factors such as basic-fibroblast growth factor, EPO was found to stimulate proliferation of neural progenitor cells, and the proliferative effects of EPO and basic-fibroblast growth factor were not additive [111]. In human, EpoR expression with associated EPO signaling was reported in the fetal central nervous system [112]. EPO and EpoR expression were observed by 7 weeks in spinal cord and found to increase between 8 and 24 weeks of human brain development [113]. EpoR expression was observed to decrease later in development, and in adult brain, it was 100-fold lower than EpoR level in adult bone marrow [114]. During development, EPO was reported in the fetal central nervous system, in the spinal fluid of normal preterm and term infants [113,115] and in neonates in the cerebrospinal fluid that was elevated with hypoxia and with intraventricular hemorrhage, and did not correlate with plasma EPO concentration with EPO treatment, providing further suggestion that EPO does not cross the blood–brain barrier [116]. In adults, EPO level in cerebrospinal fluid was observed to be 30-fold lower than serum levels in patients with traumatic brain injury [99].

*EpoR^−^*^/*−*^ mice die *in utero* at day E13.5 and exhibit a thinner neuroepithelium; brain development is affected as early as E10.5, due to reduction in neural progenitor cells and increased apoptosis [45]. In contrast, no gross morphologic changes are observed in adult mice with EpoR restricted to erythroid tissue [84]. However, an intrinsic defect in neurons of increased susceptibility to ischemia in embryonic *EpoR^−^*^/*−*^ mice was observed in neurons from embryonic mice with selective EpoR expression driven by the endogenous *EpoR* promoter in hematopoietic/endothelial tissue but not in brain, which rescues the severe anemia associated with *EpoR^−^*^/*−*^ mice [111]. These mice as well as mice with brain specific deletion of EpoR survive through adulthood with no gross morphological defects, although in adult brain, neural cell proliferation and viability were reduced with increased susceptibility to glutamate damage and stroke damage [105,111].

Functional activity for EPO production in brain was proposed in studies in rodents that blocked endogenous brain EPO activity. With direct infusion to the lateral ventricle of gerbils of soluble EpoR extracellular domain capable of binding EPO, mild ischemia caused impaired learning ability and neural degeneration with increased apoptosis in hippocampal CA1 neurons that was not observed in control animals or with infusion of denatured protein [117]. Conversely, preconditioning with EPO infusion to the lateral ventricle was observed to reduce ischemia-induced learning disability and ischemic death of CA1 neurons [117]. The neuro activity of endogenous EPO was suggested by the increased susceptibility to glutamate toxicity in mice that lack EpoR in brain, and *in vitro* by the reduction in glutamate toxicity and increased neuronal survival in the absence of neurotropic factors with EPO treatment in hippocampal and cortical neuron cultures from wild type mice [44,45,111]. Hypoxic preconditioning of mice by exposure to sublethal low levels of oxygen tension 24 hours prior to focal permanent ischemia showed reduction in infarct volumes [118]. This preconditioning was proposed to be a result of HIF-stimulated increase in EPO and VEGF, which in combination appear to stimulate both neurogenesis and angiogenesis [119]. Direct infusion of soluble EpoR in the brain to inhibit EPO signaling was observed to block hypoxic preconditioning and reduce the protective effect by 40%–88% [107,120]. EPO was also reported to contribute to hypoxic post-conditioning in a mouse model of cerebral ischemia [121]. The possibility that the protective effects observed in these animal models with direct brain infusion may be indirect is suggested by the low level of *EpoR* mRNA expression in adult whole brain, which is consistent with the inability of human EpoR antibody to detect EpoR protein in human brain [74]. In contrast, this human EpoR antibody readily demonstrates EpoR expression on erythroid progenitor cells, although it does not detect EpoR protein in bone marrow [74]. Evidence for a direct neural response to EPO is suggested by reports of significant EpoR expression in localized regions of the brain such as the hippocampus, subventricular zone and hypothalamus and EPO associated neurogenesis in adult mice in the hippocampus and subventricular zone [105,111,122].

In rodent models of ischemic injury in brain, *EpoR* mRNA expression was reported to increase in proximity the lesion in associated neural cells, endothelial cells, reactive astrocytes, microglia and monocytes [118,123,124], and direct EPO infusion into the cerebral ventricle was observed to reduce infarct size and place-navigation disability [123,124]. In culture, EPO was observed to reduce low oxygen induced cell death in both embryonic and postnatal hippocampal neurons [45,125]. Activation of NF-κB signaling has been proposed as a mechanism for EPO mediated neuroprotection. During the “classical” activation of NF-κB, phosphorylation of inhibitor of NF-κB (IκB) by IκB kinase (IKK), leads to degradation of IκB by ubiquitylation, and results in NF-κB translocation to the nucleus. However, after EPO treatment of neural cells, JAK2 was found to phosphorylate IκB resulting in ubiquitylation-independent dissociation of IκB from the NF-κB complex, translocating NF-κB into the nucleus and inducing transcription of neuro-protective genes [126]. NF-κB is cytoprotective through the inhibitors of apoptotic protein (IAPs), which can block caspase activity through suppression of tumor necrosis factor-α (TNF-α) [127], via suppression of apoptotic JNK signaling [128] and direct activation of Bcl-x_L_ [129]. An alternate EPO heteroreceptor complex consisting of the classical EpoR and the common β-chain receptor (βcR) has been proposed for neural cells [130]. However, in the rat brain βcR does not appear to localize with EpoR or EPO and βcR is below the level of detection in EPO-responsive neuronal cell lines, SH-Sy5y and PC12 [131,132].

Direct injection of an EPO expressing viral vector into the spinal cord following cord contusion injury was reported to improve locomotor function and reduced tissue damage and inflammation, raising the possibility that early and sustained delivery of EPO following spinal cord injury in the rodent model contributes to recovery [133], although the therapeutic window of opportunity and long term effects were not established. While EPO does not readily cross the blood brain barrier, the acute reduction in injury observed with systemic EPO administration in animal models was suggested to relate indirectly to an endothelial response including improved oxygen delivery via NO production [78,134]. For example, in focal cerebral ischemia, EPO was reported to stimulate endothelial cell secretion of matrix metalloproteinase 2 (MMP2) and MMP9 via PI3K/AKT and ERK1/2 signaling pathways and enhance neural progenitor cell migration to the area of injury [134]. Systemically administered EPO was reported to reduce injury and inflammation in rat models of spinal cord compression and contusion injury [135]. However, subsequent analogous studies did not observe improvement in motor function or any other beneficial effect [136]. The role of systemic EPO administration in spinal cord injury remains uncertain as a recent study of analogous spinal cord injury reported better descending and ascending responses and higher levels of myelin with EPO treatment, most prominent at relatively early times [137].

### 5.4. Skeletal Muscle and Wound Healing

Pax-7^+^ satellite cells are involved in the growth and regeneration of skeletal muscle, can self-renew or give rise to Myf5^+^ committed muscle progenitor cells that differentiate to myoblasts, muscle precursor cells and, with terminal differentiation, fuse to form myotubes and myofibers [138]. In culture, EPO was observed to stimulate myoblast proliferation and increase expression of the basic-helix-loop-helix muscle specific transcription factors, Myf5 and MyoD, but not myogenin, required for late muscle differentiation and fusion to myotubes, as well as transcription factors GATA3, GATA4, and TAL1, and EpoR, mediated in part by induction of Sirt1; in differentiation medium EPO decreased myogenin and MCH protein, markers of myoblast differentiation [29,46,139]. Forced expression of Myf5, GATA3, GATA4 or TAL1 was found to increase *EpoR* expression and myoblast proliferation, consistent with EPO stimulated proliferation and *EpoR* expression; Myf5, GATA3, GATA4 and EpoR, along with EPO response, were down-regulated with terminal differentiation with increased expression of myogenin and myotube fusion [46]. While EpoR protein expression was reported in satellite cells of human skeletal muscle, EpoR was not detected on human mature skeletal muscle and EPO administration in healthy subjects did not invoke an acute or long-term response [140,141]. Adding to the uncertainty of direct skeletal muscle response to EPO treatment, EpoR protein was reported in mouse and human myoblasts and skeletal muscle without response to EPO treatment, while replenishment of fresh medium and not EPO treatment activated JAK2 and other signaling pathways [142].

Although EpoR expression in adult skeletal muscle may be low or below the level of detection [74,122], EPO treatment has been reported to increase recovery from skeletal muscle injury, increasing the number of Pax-7^+^ satellite cells at the site of injury by two-fold and reducing the number of damaged fibers by a half in a mouse model of cardiotoxin induced injury [143], mediated possibly via direct myoblast response or indirectly such as via improved oxygen delivery. A role for endogenous EPO in skeletal muscle repair was suggested by mice with EpoR restricted to erythroid tissue that shows no gross organ defects including skeletal muscle [84]. However, these mice had fewer skeletal muscle Pax-7^+^ satellite cells, myoblasts that do not proliferate in culture, and are more susceptible to skeletal muscle injury [143]. In comparison, transgenic mice with high EPO expression had more Pax-7^+^ satellite cells and reduced muscle injury, and wild type mice with acute EPO treatment at the time of injury exhibited increased skeletal muscle recovery without increase in hematocrit [143]. In a mouse model for cell therapy for muscular dystrophy, overexpression of *EpoR* in myoblasts and/or EPO treatment at the time of transplant was suggested to increase donor cell survival and increase the number of fibers expressing dystrophin without change in hematocrit [139]. The contribution of endothelial EPO response to skeletal muscle injury was suggested by reports of EPO treatment in rodents that protect musculocutaneous tissue from ischemic necrosis via eNOS activation and maintaining capillary perfusion [144], and in a hind limb ischemia injury model in mice that enhances blood flow recovery [145]; EPO was also observed to increase regeneration of skeletal muscle tissue after severe trauma and to improve microcirculation in muscle tissue in rat [146].

Therefore, in contrast to skeletal muscle in normal healthy individuals that appeared non-responsive to exogenous EPO administration, EPO treatment in animal models of skeletal muscle injury was suggested to stimulate skeletal muscle repair and wound healing. Whether an injured human skeletal muscle responds to exogenous EPO to facilitate muscle repair and regeneration is still undetermined. With respect to fiber type, although *ex vivo* isolated muscle fiber is not EPO responsive, EPO activity may affect fiber type specification. Young mice expressing high transgenic EPO exhibit an increase in proportion of slow twitch myofibers and expression of corresponding proteins associated with muscle fiber type, and increased mitochondrial activity while mice with EpoR restricted to erythroid tissue exhibit a decrease in slow twitch myofibers and decrease mitochondrial activity [147], suggesting that during skeletal muscle development EPO signaling may contribute to the fate decision between slow and fast twitch fiber specification.

## 6. EPO Regulation of Metabolism and Obesity

Early, small patient studies of EPO suggested possible improvement in glycemic control and insulin sensitivity with EPO treatment in hemodialysis patients or with repeated phlebotomies (accompanied by an increase in endogenous EPO level) in diabetic patients for autologous transfusion, and improved glycemic control in diabetic patients undergoing repeated phlebotomies prior to surgery [148,149,150]. Investigation of metabolic response in animal models provides insight into potential EPO regulation of metabolic homeostasis.

### 6.1. Phenotype of Mice with EpoR Restricted to Erythroid Tissue

**Figure 4:**
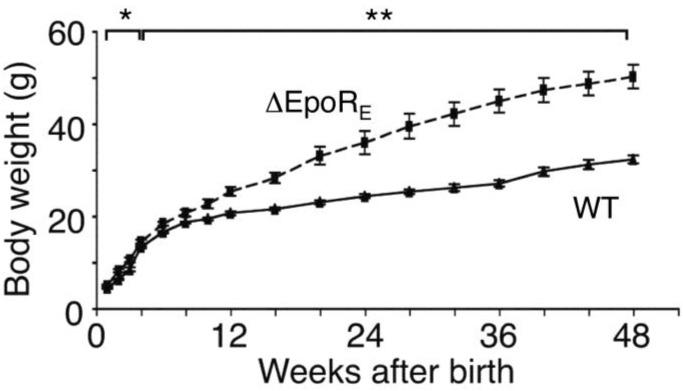
EpoR expression in non-hematopoietic tissues regulates body weight gain. Female mice with EpoR restricted to erythroid tissue (Δ*EpoR_E_*) gain more body weight compared to wild-type (WT) control, starting as early as 4 weeks of age, and the effect is considerably more prominent with advancing age. * indicates *p* < 0.05; ** indicates *p* < 0.01. Reprinted from [122] with permission. Copyright 2011 Nature Communication.

*EPO^−^*^/*−*^ and *EpoR^−^*^/*−*^ mice die of severe anemia around embryonic day 13.5 [2,3]. Expression of an erythroid-specific *EpoR*-transgene driven by GATA-1 hematopoietic regulatory domain (GATA-1-HRD) rescues mice (Δ*EpoR_E_*) on an *EpoR^−^*^/*−*^ background and restores normal erythropoiesis [84]. Although these mice have no apparent gross abnormal morphology, body weight increases from the first week after birth and the mice became obese, evident by 4 months in female mice (Figure 4) and 6 months in male mice, attributed to increase in fat mass but not lean mass [122]. In addition to the obese phenotype, Δ*EpoR_E_* mice became insulin resistant with age, and exhibited decreased energy expenditure and motor activity via effects on white adipose tissue and hypothalamus.

*EpoR* mRNA is expressed in white adipose tissue (WAT) in mice at 60% the level of hematopoietic tissue and *EpoR* expression is comparable in both adipocyte and stromal vascular cell fractions in lean and obese mice [72,122]. Adipocyte size distribution in gonadal adipose tissue shifted to smaller cells in Δ*EpoR_E_* mice with lower WAT phosphorylation of p38MAPK, ERK44, ERK42 and PPARγ [122], supporting a role for EPO in preadipocyte differentiation. EpoR expression in murine hypothalamus was observed to localize to proopiomelanocortin (POMC) neurons and POMC expression was decreased in Δ*EpoR_E_* mice [122]. Furthermore, EPO treatment in wild type mice was found to increase hypothalamus expression of POMC; expression of other hypothalamic neuropeptides, such as agouti-related protein (AGRP), neuropeptide Y (NPY), melanin-concentrating hormone (pro-MCH) or prepro-orexin, were not affected by EPO treatment or in Δ*EpoR_E_* mice. These findings suggest a central regulatory role of EPO in energy homeostasis via its target in arcuate nucleus of the hypothalamus.

### 6.2. EPO Treatment in Genetic Mouse Models of Obesity

Leptin, a 16 kDa hormonal product of the obesity (*ob*) gene, is primarily secreted by adipocytes and regulates food intake and energy homeostasis mainly through its action on the hypothalamus and mice genetically deficient in leptin (*ob*/*ob*) or its receptor (*db*/*db*) become grossly overweight, hyperphagic and develop severe insulin resistance [151]. EPO and leptin share some structural similarities as members of the class-I cytokine superfamily and both signal through JAK/STAT pathways. EPO treatment in *db*/*db* mice was reported to promote VEGF expression, which could stimulate angiogenesis, and improved wound healing, stimulated podocyte survival, and, particularly with low dose administration, reduced albuminuria and diabetic kidney damage [152,153,154]. EPO treatment in *db*/*db* mice was also reported to reduce damage in cardiomyopathy, inhibiting transforming growth factor-β (TGF-β) and activating AKT signaling pathways as observed in kidney, and reduce injury to insulin producing pancreatic β-cells via antiapoptotic, anti-inflammatory and angiogenic effects in the islets [154,155,156].

Leptin treatment in *ob*/*ob* but not *db*/*db* mice reduced body weight, percentage of body fat, food intake, and serum concentration of glucose and insulin [157,158,159]. Interestingly, EPO treatment in *ob*/*ob* mice was observed to improve overall metabolic characteristics, reduce blood glucose levels, improve glucose tolerance and attenuate weight gain, without affecting food intake [160]. While EPO treatment of *ob*/*ob* mice increased hematocrit, the reduction in weight gain, specifically in fat mass, was found even when hematocrit was maintained at normal levels by phlebotomy, suggesting that the increased erythropoietic load was not responsible for this effect [122].

### 6.3. Mice with Chronic Elevation of EPO

Transgenic mice generated using a human platelet-derived growth factor B-chain promoter to drive human *EPO* cDNA (*tg6*), express high circulating EPO (12-fold) [80] and are a useful model for chronic EPO administration. These mice have high hematocrit (79%), and exhibit lower blood glucose and serum insulin levels, improved glucose tolerance and insulin sensitivity, and lower body weight and fat mass [160]. These mice were also observed to have a greater number of Pax-7^+^ satellite cells in skeletal muscle and improved repair and regeneration following skeletal muscle injury [143].

The *tg6* mice have an increased respiratory frequency but not a stimulate hypoxic ventilatory response [161]. In contrast, transgenic mice with chronic elevated transgenic EPO expression in brain with normal circulating EPO level and hematocrit (*tg21*) were observed to have improved ventilatory response and acclimatization to severe acute and chronic hypoxia [162]. Transgenic *tg21* mice were also reported have improved exercise performance with increased maximal aerobic capacity and prolonged time to exhaustion that was also observed in mice receiving a single bolus injection of high dose EPO that did not increase erythroid response [163]. Intrathecal EPO administration at midcervical C4 in rats was reported to contribute to neural system respiratory motor control and elicited long lasting phrenic motor facilitation mediated via ERK and AKT activation [164]. These rodent models provide evidence for EPO regulation of energy homeostasis and EPO contribution to central regulation of physical activity and respiration.

### 6.4. Fat Specific Deletion of EPO Receptor (EpoR)

**Figure 5:**
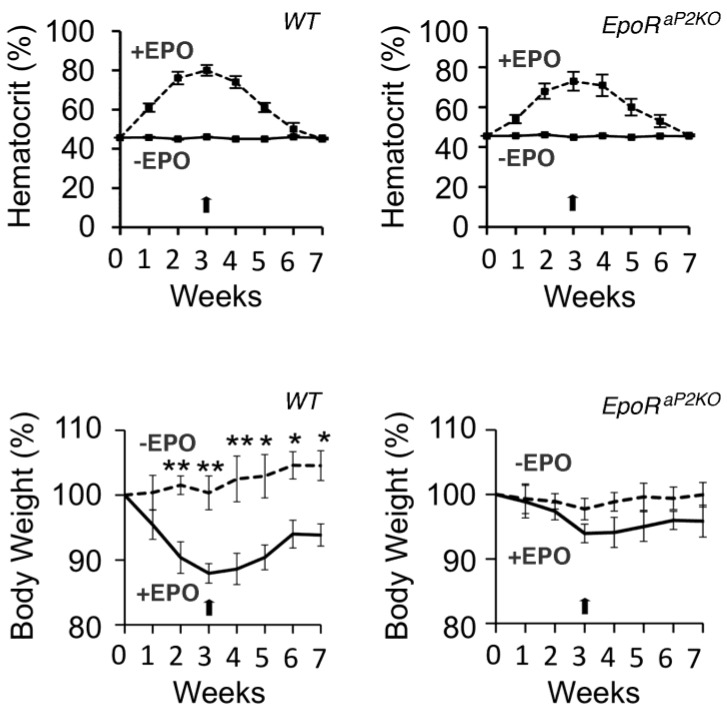
EPO treatment reduces body weight independent of the increased hematocrit. **Left panels**, wild type (*WT*) mice (C57Bl/6 background) treated with EPO for 3 weeks (3000 U/kg; 3 × weekly) show the characteristic increase in hematocrit and decrease in body weight. At the end of EPO treatment (arrow), hematocrit returns to normal by 4 weeks with a more gradual normalization of body mass; **Right panels**, in mice that lack EpoR in white adipose tissue (*EpoR^aP2KO^*) treated with EPO show the increase in hematocrit without the significant decrease in body weight, demonstrating that the change in hematocrit is not sufficient to cause the decrease in body weight and the change in fat mass requires EpoR expression in white adipose tissue. * indicates *p* < 0.05; ** indicates *p* < 0.01. Reprinted from [30] with permission. Copyright 2013 Diabetes.

Mice with EpoR restricted to erythroid cells resulting in increased body weight and white fat accumulation drew attention to the metabolic activity of EPO [122]. In contrast, EPO treatment in wild type mice, EPO over production in skeletal muscle and mice expressing high transgenic levels of EPO were observed to have reduced body weight and adipose tissue mass, improved glucose tolerance and reduced insulin levels compared with control mice [122,160,165]. In addition, the level of EpoR expression in white adipose tissue (WAT), which is secondary to its primary expression site of EpoR in erythroid progenitor cells [122], suggested the possibility that endogenous EPO action in WAT protects against obesity to maintain energy homeostasis. Fat specific deletion of EpoR (*EpoR^aP2KO^* generated by mating *EpoR*-floxed mice and *aP2-Cre* mice on a C57Bl/6 background as a model system for diet induced obesity) preferentially increased body weight and fat mass, and by 30 weeks these mice had 65% greater fat mass with reduced oxygen consumption and total respiratory exchange ratio [30]. On a high fat diet, these mice were observed to have greater increase in fat mass, increased glucose intolerance and insulin resistance. Wild type mice treated with EPO exhibited the expected increase in hematocrit and a significant decrease in body weight, while *EpoR^aP2KO^* mice exhibited the increase in hematocrit but no significant reduction in body mass (Figure 5). In an analysis of WAT, loss of EpoR was observed to decrease in mitochondrial biogenesis, cellular oxygen consumption and fatty acid metabolism in *EpoR^aP2KO^* mice, and suggested that endogenous EPO/EpoR activity in WAT contributes to the energy-sensing network of PPARα-Sirt1-PGC-1α [30]. Although indirect effects of EPO on whole body metabolism cannot be entirely excluded, the *EpoR^aP2KO^* mouse model with fat specific knockout of EpoR suggested that the loss of EPO/EpoR signaling in adipose tissue is sufficient to develop the metabolic syndrome phenotype including obesity, glucose intolerance and insulin resistance and supports the idea that EPO activity in fat may contribute to energy homeostasis. Of note, in addition to deletion of EpoR in fat cells in C57BL/6 mice, a strain commonly used as a model of high fat diet induced diabetes [166,167,168], EpoR was also deleted in fat cells in mice on a mixed background [169]. These mice were metabolically similar to control mice, although no evidence was provided for EPO metabolic activity in the control mice. The effect of background strain on obesity and EPO metabolic response suggests the confounding variability of genetic background not only on susceptibility to obesity but also to metabolic response to EPO.

### 6.5. Pancreatic Islet Cell Response in Mice Treated with EPO

Insufficient functional pancreatic β-cell mass is one of the pathogenic mechanisms of type 1 and 2 diabetes that leads to the suggestion that promoting β-cell growth and survival may be helpful for diabetes prevention and treatment. EPO has been demonstrated to be cytoprotective in select non-erythroid cells that express EpoR. In rodents, EPO protects against streptozotocin (STZ) induced type 1 diabetes by reversing STZ-mediated β-cell destruction, an activity that is abrogated with EpoR knockout in pancreatic islet, and is protective against diabetes in the db/db mouse model of type 2 diabetes [156,170]. EPO protection is suggested to be mediated via stimulated increase in angiogenesis as indicated by induction of c-Myc, c-kit and VEGF expression, and β-cell mass, rather than direct EPO effects on β-cell function [156].

### 6.6. Metabolic Response to EPO Treatment in Patients

In rodent studies, EPO treatment was found to increase hematocrit and reduce body fat in obese animals. While genetic manipulations in mice provide some insight into EPO contributions to metabolic homeostasis, such as potential response of white adipose tissue, hypothalamus and pancreas, small patient studies suggest that EPO administration may also affect human metabolism. Since EPO was first introduced into the clinic in 1989, it has been used as an effective agent to treat anemia associated with chronic renal failure and cancer for two and half decades. From recombinant human erythropoietin (rHuEPO) epoetin to darbepoetin alfa and continuous erythropoietin receptor activator (CERA), various therapeutic agents have been developed to stimulate erythropoiesis with greater metabolic potency and longer half-life [1,171,172]. In recent years, studies assessed benefit of EPO administration in metabolic regulation, neuroprotection, muscle performance, and cardioprotection.

EPO treatment in humans was evaluated for improved insulin sensitivity and lipid profile. For example, a study of 33 hemodialysis patients reported that EPO treatment either intravenously or subcutaneously three times per week for one year positively affected the lipid profile at the end of each dialysis [173]. However, another study of 102 maintenance hemodialysis patients who were treated for 2 years with EPO given intravenously at the end of the dialysis session did not demonstrate an EPO effect on lipid metabolism [174]. In indirect calorimetry analysis of healthy human subjects, a single EPO injection was reported to not affect glucose metabolism or whole body protein metabolism, but significantly increased resting energy expenditure [175]. Hence, the metabolic effects of EPO treatment in patients are confounding and may vary according to the duration, dose and health status of individuals.

## 7. EPO Regulation of Inflammation

Inflammation can affect EPO response while EPO, in turn, can modulate inflammation. For example, during inflammation bone marrow erythropoiesis in mice is suppressed while the spleen provides the response to EPO stimulation and this stress erythropoiesis appears to be mediated via induction of bone morphogenic protein 4 by spleen macrophages [176]. On the other hand, EPO can exert direct and indirect influence on inflammatory response in animal models of ischemic and traumatic injury including reduction of immune cell response, inhibition of proinflammatory cytokine production and stromal and endothelial cell response.

### 7.1. Central Nervous System

In rodent models of ischemic brain injury and traumatic brain injury, EPO treatment was suggested to reduce neuronal death and associated astrocyte activation, leukocyte and microglia recruitment to the site of infarction or injury, and proinflammatory cytokine production [177,178]. In experimental intracerebral hemorrhage, EPO reduction of inflammation and apoptosis was coupled with activation of eNOS, STAT3 and ERK [69]. In a rat model of autoimmune neuritis, EPO reduced disease severity, inflammation and T-cell infiltration, while increasing macrophage number toward an anti-inflammatory phenotype and TGF-β production within the peripheral nerve local environment [179]. An anti-inflammatory response to systemic EPO administration was reported in rat models of spinal cord compression and contusion injury [135], although subsequent studies did not demonstrate a beneficial effect [136]. However, direct expression of EPO by viral vector into the spinal cord following cord contusion injury was reported to reduce tissue damage [133].

### 7.2. Cardiovascular

In the cardiovascular system, EPO pretreatment was suggested to reduce a systemic inflammatory response in myocardial ischemia-reperfusion injury in rodents mediated via PI3-kinase activation of AP1 and NO production by eNOS [95]. With EPO preconditioning in a rat model of cardiac ischemia-reperfusion injury, the observed reduction in infarct size was accompanied by a reduction in inflammatory cytokines, TNF-α and interleukin 6, and NF-κB [180]. In autoimmune disease, EPO treatment in a rat model of experimental autoimmune myocarditis was reported to reduce the area of inflammation, macrophage and CD4 T cell infiltration in the myocardium, and expression of inflammatory cytokines [181].

### 7.3. Gut and Liver

In inflammatory bowel disease with anemia refractory to treatment with iron and vitamins, EPO administration in combination with oral iron improved anemia [182]. In chemically induced colitis, EPO treatment was suggested to inhibit induction of activated macrophages and expression of proinflammatory genes such as inducible NO synthase (iNOS/NOS2), TNF-α, interleukin 6, and blocks NF-κB activation to limit tissue damage in the gut [183]. EPO treatment was reported to reduce inflammation in the intestine in traumatic brain injury in rats, reducing inflammatory cytokines, TNF-α and interleukin 8 as well as labile zinc accumulation [184], and to reduce injury in liver in rodents in ischemia-reperfusion injury or in high fat diet induced obesity, inhibiting gluconeogenesis and inflammation [185,186].

### 7.4. Macrophages

Macrophages are leukocytes with key innate immune functions with phagocytic and anti-microbial activities, and include tissue-resident macrophages such as splenic macrophages, kupffer cells (liver), microglia (brain), osteoclasts (bone) and adipose tissue macrophages [187]. Macrophages have an important role in promoting erythropoiesis and also contribute to tissue surveillance, homeostasis, stress response and wound healing, and adipose tissue macrophages have an important function in metabolic disorders [188,189]. EPO was suggested to reduce macrophage lipid accumulation and foam cell formation in a mouse model of atherosclerosis [190]. In mouse models of infection and colitis, EPO activation of EpoR on macrophages was reported to increase JAK2 signaling, inhibit NF-κB p65 signaling pathway and interfere with innate immune response resulting in increased deterioration of a *Salmonella* infection mouse model for sepsis but ameliorating chemically induced colitis in mice [183]. Lastly, endogenous and exogenous EPO has been found to exert anti-inflammatory effects on white adipose tissue macrophages during diet induced obesity that was exacerbated in mice with EpoR restricted to erythroid tissue, thus implicating the EPO/EpoR axis in regulation of macrophage infiltration and subset composition in white fat [72].

## 8. EPO Therapy and Associated Adverse Events

Clinical studies of EPO administration including efforts to increase hematocrit to the normal range as well as treatment of non-anemic conditions such as stroke or cardiovascular disease, revealed adverse consequences with EPO therapy and suggest caution and consideration be given to optimum dose and route of EPO administration, the timing of EPO treatment relative to onset and extent of injury, and potential confounding factors of other treatment protocols.

### 8.1. Chronic Kidney Disease

Recombinant human EPO is approved for treatment of anemia resulting from chronic kidney failure, chemotherapy and antiviral treatment for Human Immunodeficiency Virus (HIV), and to reduce blood transfusions associated with selected major surgeries. The association of anemia with greater risk including cardiovascular disease in chronic kidney disease led to several clinical trials designed to increase hemoglobin in patients with chronic kidney disease. In the Cardiovascular Risk Reduction by Early Anemia Treatment with Epoetin Beta (CREATE) and Correction of Hemoglobin and Outcomes in Renal Insufficiency (CHOIR) trials, the high hemoglobin target group (13.0 and 13.5 g/dL, respectively) did not correlate with reduced risk of cardiovascular events, but did correlate with increased dialysis requirement (CREATE) and was associated with more adverse events of myocardial infarction, congestive heart failure, stroke and death (CHOIR) compared with the low hemoglobin target group (in the range of 10.5–11.5 g/dL) [191,192]. In the Trial to Reduce Cardiovascular Events with Aranesp Therapy (TREAT) (patients with type 2 diabetes not on dialysis), the high hemoglobin target group (13 g/dL) received fewer red cell transfusions but correlated with a greater risk of stroke and no reduction in cardiovascular event or death compared to the low hemoglobin target group (at least 9 g/dL) [193]. The increased risk in high hemoglobin targets with EPO treatment observed in these trials led to more conservative recommendation for dosing in EPO treatment by the U.S. Food and Drug Administration (FDA) in 2011. The recommended target hemoglobin level was reduced from the range of 10–12 g/dL to hemoglobin levels less than 10 g/dL for chronic kidney disease.

In secondary analysis, the CHOIR trial revealed better outcomes in patients achieving their target hemoglobin (high or low) than patients who did not [194], suggesting that patients who demonstrate appropriate response to elevated EPO levels are associated with better outcome. Furthermore, additional analysis of the TREAT trial of patients with kidney disease and type 2 diabetes suggested that a poor initial response to EPO was associated with a lower hemoglobin level at 12 weeks, higher EPO dose and increased risk of cardiovascular events or death [195]. The CHOIR trial also revealed increased risk or significantly greater hazard in patients with the higher hemoglobin target that was not seen in patients with diabetes or heart failure, indicating that comorbidities differentially affect outcomes in EPO treatment of anemia [196].

### 8.2. EPO and Cancer

In cancer, EPO treatment for anemia resulted from successful clinical trials of patients with anemia associated with chemotherapy and with lymphoproliferative malignancies where EPO administration reduced transfusion requirements, increased hemoglobin concentration and decreased fatigue and improved quality of life [197,198,199]. In contrast to these trials, later studies to normalize hemoglobin levels demonstrated decreased benefit and survival, and increased thrombotic and vascular events, particularly in breast and head and neck cancers [200,201]. An overall analysis of clinical trials for EPO treatment of anemia associated with chemotherapy in nonmyeloid cancer patients revealed the increased risk of venous thromboembolism and mortality with EPO treatment in select solid cancers [202], calling attention to the potential deleterious effect of efforts to elevate hemoglobin level into the normal range and resulting in lowering recommended targets for EPO therapy to treat anemia in cancer patients [203].

### 8.3. Stroke Study

A few clinical studies suggested the possibility of potential benefit of EPO treatment for neurologic disease. A meta-analysis on all of the 180 preclinical studies reported during the previous 12 years yielded positive results on EPO as a neuroprotective drug [204]. The neurologic benefit from EPO treatment as supportive therapy has been suggested specifically for acute stroke [205], chronic schizophrenic patients [206,207], patients with type 1 diabetes mellitus [208] and carbon monoxide poisoning [209], and for mood symptom and depression associated memory dysfunction possibly relating to hippocampus-dependent memory [210].

A pilot study in acute stroke patients suggested increased cerebrospinal fluid EPO with intravenous administration, an association with improved outcome and a trend for reduction in infarct size observed by magnetic resonance imaging (MRI) [205]. However, a follow-up Phase II/III clinical trial (German Multicenter EPO Stroke Trial) of 460 patients was negative for the beneficial effects of EPO, EPO treatment correlated with increased risk of serious complications including intracerebral hemorrhage, thromboembolic events and death, especially and unexpectedly in patients also receiving recombinant tissue plasminogen activator (TPA) for thrombolysis to improve clinical outcome of patients with early onset of symptoms [211]. Nevertheless, exploratory subgroup analysis of the German Multicenter EPO Stroke Trial suggested a clinical benefit from EPO treatment in patients not receiving thrombolysis [212].

Recent phase I/II trials were conducted to assess the safety and efficacy of EPO neuroprotection and, although they may be underpowered, these trials did not correlate with an increase in adverse events or a benefit from EPO treatment compared with placebo groups [213,214,215]. In one study, 59 neonates undergoing congenital heart surgery were assessed for safety, including magnetic resonance imaging for brain injury, and for cognitive, language and motor scores [213]. In another trial, 50 patients scheduled for coronary artery bypass graft surgery treated were treated with single large dose of EPO or placebo before cardiopulmonary bypass and assessed for cardiac and cerebral ischemic blood markers and pro-inflammatory markers [214]. A trial that was halted early after enrollment of 90 patients with acute ischemic stroke was designed to assess combined treatment of human choriogonadotrophin on days 1, 3 and 5 with EPO treatment on days 7, 8 and 9 [215]. The potential benefit in neurologic response of EPO treatment suggested by studies in animal models of diseases has not yet been translated to successful phase III clinical trials.

### 8.4. Cardiovascular Studies

Preliminary patient studies suggested the potential of EPO treatment for acute myocardial infarction before or during percutaneous coronary intervention [216,217]. However, larger studies did not demonstrate a reduction in infarct size with EPO treatment [218,219,220,221]. The Reduction of Infarct Expansion and Ventricular Remodeling With Erythropoietin After Large Myocardial Infarction (REVEAL) trial included patients with ST-segment elevation myocardial infarction (STEMI) with successful percutaneous coronary intervention or rescue reperfusion strategy and utilized EPO treatment within 4 h of reperfusion, demonstrated no reduction in infarct size and was associated with higher rates of adverse cardiovascular events [218]. In rodent studies, EPO protection in heart ischemia or ischemic/reperfusion injury indicates the importance of EPO dosage and timing from the onset of ischemia or ischemia/reperfusion injury and suggests that even in the rat model, EPO administration does not reduce myocardial infarct size when administered 6 h or later from the initial time of occlusion, as would be the case in the REVEAL trial [222,223]. In other trials with a single dose of EPO administered after reperfusion with STEMI, reduction in infarct size was also not observed, although when EPO was administered immediately after reperfusion, outcome was favorable in secondary end points such as left ventricular volume, but not when delayed and administered within 3 h of reperfusion, and the possibility of increased adverse events was also suggested in another trial [219,220,221]. As with patient studies of EPO administration in chronic kidney disease and stroke [195,196,211], comorbidities and concomitant treatments such as a clot-busting (thrombolytic) drug for reperfusion therapy are also likely to influence responses to EPO treatment in myocardial infarction.

## 9. Conclusions

**Figure 6:**
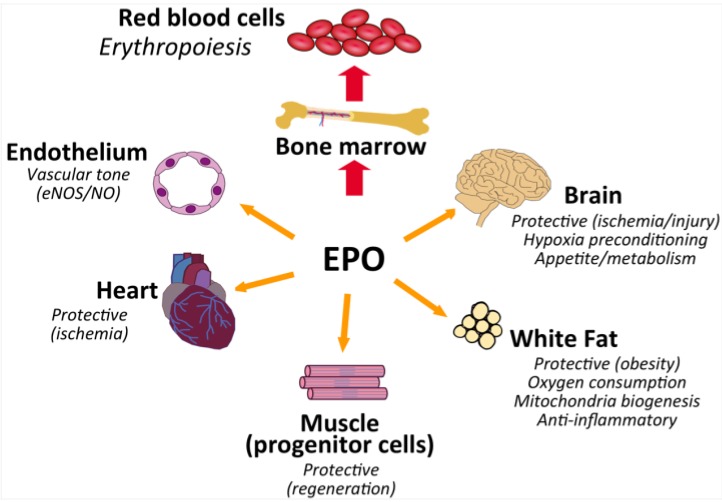
Pleiotropic effects of EPO. EPO is a multi-functional cytokine with the primary activity to regulate red blood cell production in the bone marrow. EpoR, expressed at the highest level in erythroid progenitor cells, is also expressed in select non-hematopoietic tissues that provides for EPO response. Non-hematopoietic expression of EpoR and the potential consequence of EPO activity gleaned from model systems include vascular endothelium to stimulate NO production to regulate vascular tone, heart to provide protection against ischemic injury, skeletal muscle myoblasts to promote wound healing, white adipose tissue for metabolic regulation and protect against diet induced obesity, and brain for metabolic regulation and to provide protection against injury.

EPO induction at low oxygen increases red blood cell production, which increases oxygen delivery in response to ischemic stress. EPO responses in several animal and cell culture models of ischemic stress and injury were observed to provide benefit beyond red blood cell production during development, maintenance or repair (Figure 6), mediated directly via tissue specific response or indirectly such as through improved oxygen delivery. The mature erythroid form of EpoR was reported in several non-hematopoietic cells and tissues, albeit at lower levels than in erythroid progenitor cells. Nevertheless, observations in genetically modified mice with targeted EpoR expression in non-hematopoietic tissue including brain and white adipose tissue suggest potential benefit of EPO response beyond red blood cell production.

Mice with EpoR restricted to erythroid tissue survive to adulthood with no gross morphological abnormalities, providing evidence that EpoR is required only for red blood cell production, although these mice exhibit increased susceptibility to stress response such as pulmonary hypertension, skeletal muscle injury, and inflammation in diet induced obesity, and these mice become obese, glucose intolerant and insulin resistant. Endothelial response to elevated EPO was further suggested in transgenic mice expressing high human EPO with resultant increased eNOS activity and NO production offsetting the elevated hematocrit, and also in mice with EpoR restricted to hematopoietic and endothelial tissue in which acute EPO administration stimulated eNOS activity and reduced heart ischemia reperfusion injury. Reduced neurogenesis in adult brain and increased susceptibility to stroke damage were observed in mice that lack EpoR in brain, and mice with targeted deletion of EpoR in white adipocytes were reported to be more susceptible to diet induced obesity. While the question remains about how a low level of EpoR can provide a direct EPO response, various responses to EPO observed in animal and cell models of ischemic stress, injury or metabolism in non-hematopoietic tissues suggest therapeutic benefit for EPO beyond anemia.
